# Special Issue “Mechanical Performance of Sustainable Bio-Based Compounds”

**DOI:** 10.3390/polym14224832

**Published:** 2022-11-10

**Authors:** Antonella Patti, Domenico Acierno

**Affiliations:** 1 Department of Civil Engineering and Architecture (DICAr), University of Catania, Viale Andrea Doria 6, 95125 Catania, Italy; 2 Regional Center of Competence New Technologies for Productive Activities Scarl, Via Nuova Agnano 11, 80125 Naples, Italy

The global production of plastic is increasing, and plastic represents one of the most popular materials, widespread in countless applications in commercial and industrial fields and everyday life. However, the main drawback to consider in this widespread employment is the difficulty of properly disposing of plastic at the end of its life cycle, and its consequent accumulation in nature. Plastic wastes, including microplastics (1 to 5000 µm particles), accumulate in the oceans, seriously altering the marine environment and the lives of its inhabitants. Furthermore, millions of tons of CO_2_ are emitted into the atmosphere, generated from both the production processes and the final incineration at the end of the products’ life cycles. The huge emission of greenhouse gases such as CO_2_ further contributes to global warming by inducing dramatic and irreversible weather changes [1].

In this context, scientific research should focus on alternative solutions for the sustainable development of production processes, including biomass waste valorization [2] and the use of green technologies, avoiding risks for human health, and limiting the environmental impact. For example, fruit and vegetable wastes have high water contents and high concentrations of biodegradable organic substances (e.g., carbohydrates, lipids, and organic acids). These can be efficiently recovered and valorized as sources of polymer materials for active packaging [3]. Innovative technologies, involving both thermal and non-thermal methods, have been applied for the extraction and fortification of sensitive bioactive compounds, as reported in a recent review by Basri et al. [3].

The purpose of this Special Issue was to collect original contributions, both research papers and reviews, showing recent results and/or positive advances in the behavior of new sustainable biomaterials under applied mechanical stress, in both static and dynamic modes, and evaluating the characteristics of resistance, moduli and/or viscoelasticity in view of potential applications in biotechnology and biomedicine [4], tissue engineering [5], medical devices [6,7], surgical or dental implants [8], agriculture [9], packaging [3,10,11], textiles [1], automotives [12,13,14], green buildings and construction [15], radiation dosimetry [16], and 3D printing [17,18,19].

Wound-care products have been prepared by using gellan gum and virgin coconut oil (VCO) and developing microemulsion-based hydrogels [7]. The effects of drying methods on carboxymethyl cellulose and citric acid coating layers on cotton threads have been examined in [6], with regard to wound-dressing applications.

Arboblend V2 Nature biopolymer (which features lignin as a basic matrix; a significant amount of polylactic acid; and small amounts of natural additives such as resins, waxes, and shellac) was covered with three ceramic powders: Amdry 6420 (Cr_2_O_3_), Metco 143 (ZrO_2_ 18TiO_2_ 10Y_2_O_3_), and Metco 136F (Cr_2_O_3_-xSiO_2_-yTiO_2_). These systems have been considered suitable for operating in harsh conditions, such as in the automotive industry, to replace plastic materials, in light of their adhesion strength; microindentation hardness; and thermal, structural, and morphological properties [12].

Bio-based fibers can be efficiently applied in packaging when the mechanical properties of the corresponding fiber materials exceed those of conventional paperboard. Hot-pressing has been considered an efficient method for improving both the wet and dry strength of lignin-containing paper webs. Different pressing conditions for webs formed with thermomechanical pulp have been studied by Mattsson et al. [11].

Lignin and soy flour have been proposed as binders in the fabrication of *Rhizophora* spp. particleboard, for use as a phantom material in radiation dosimetry applications [16]. An increased internal bond strength was confirmed in samples with binders, which may indicate better structural integrity and physicomechanical strength.

A new sustainable food system based on rice bran biopolymers, in light of the potential application of ultrasound technology for enhancing the production of resistant starch, was proposed in [20].

An overview of recent potential applications of biopolymers in textiles was provided by Patti and Acierno [1]. Here, the use of biopolymers in various textile processes, from spinning processes to dyeing and finishing treatment, is proposed as a possible solution for reducing the environmental impact of the textile industry.

Bio-based materials have been considered a broad class of organic components ranging from agro-food renewable resources [3] (e.g., polysaccharides and proteins), bacterial activities (e.g., polyhydroxyalkanoates [21]), the conventional synthesis of bio-derived monomers (e.g., polylactides [19] and polyglycolides), or synthetic monomers (e.g., polycaprolactones, polyesteramides, bio-polyester, etc.).

These systems allow current needs to be met without dangerously burdening the future from ecological, economic, and human points of view (sustainability).

Biomaterials can be converted into chemical elements through the action of biological or physical agents (biodegradability) and can also be transformed into natural fertilizers for agriculture, once degraded (compostability).

A schematization of four types of plastics (fossil-based and non-biodegradable plastics, fossil-based and biodegradable, natural-based and non-biodegradable, and natural-based and biodegradable) is provided in [1].

However, applications of biodegradable polymers to replace conventional fossil-based plastics remain limited by the former’s poor water resistance, high-dimensional stability, and processability [22]. Most biodegradable systems have been made from polyester. The main drawback of these biopolymers is their absorption of moisture, which gives rise to dangerous degradation phenomena. In this perspective, Titone et al. [9] investigated the effect of moisture on the processing and mechanical properties of a biodegradable polyester used for injection molding. The mechanical properties of sheep wool fibers under the impact of humid air and UV irradiation have been analyzed in [23].

The addition of different fillers can effectively improve the performance of biopolymers by allowing the functional properties and degradation rate to be controlled [22]. There have been various attempts to improve the tensile, flexural, hardness, and impact strength of bioplastics by incorporating reinforcement materials, such as inorganic and lignocellulosic fibers [24]. PLA/boehmite composites were prepared using a twin-screw extruder by investigating the effects of the modification of polylactide using dicumyl peroxide as a crosslinker and Joncryl (a patented, multifunctional, reactive polymer with improved thermal stability/chain extenders for specific food-contact applications) as a chain extender on a boehmite distribution [19]. Hernandez et al. investigated the effects of microspheres, carbon fibers, or polyethylene glycol added to polyhydroxybutyrate (PHB) resin on the thermal and mechanical properties of the final compounds [21]. A polymer film based on poly(lactic acid) (PLA) as a matrix and polyaniline (PAni) as a conductive filler was prepared by Wong et al. [10], to obtain antistatic properties. Polyaniline was chosen due to to its good biocompatibility and conductivity and excellent performance in combination with a PLA matrix. The crazing technique has been applied to enhance the polymer-degrading activity of enzymes.

For specific applications, it is important to prevent alterations upon contact with biological fluids, reactions with the human body, and the release of harmful species (biocompatibility). Zirconium oxide nanoparticles (ZrO_2_ NPs) have been recognized for their high biocompatibility and resistance to wear and corrosion. El-Tamimi et al. [8] investigated the effects of adding salinized nano ZrO_2_ particles on the microstructure, hardness, and wear resistance of acrylic denture teeth.

Sustainable compounds can also be obtained from natural lignocellulosic fibers [14,17,18,25] or plant residues [15,26]. Birch (*Betula pendula* Roth.) and beech (*Fagus sylvatica* L.) solid wood and plywood were overmolded with polyamide 6 and polypropylene (PP) to investigate the mechanical properties and interfacial adhesion in [14]. Wood flour and wood pellets manufactured from secondary processing mill residues, originating from white cedar, white pine, spruce-fir and red maple, were added to polypropylene, in the presence and absence of the coupling agent maleic, to evaluate the tensile, impact, and flexural strength [25]. Wood flour, supplied from a wood-processing factory, was added to recycled polyethylene that was made by regenerating films from greenhouse covers, to investigate the rheological responses of the final materials [18]. Additionally, 3D-printed objects have been prepared by depositing a PLA-fused filament onto jute fabrics and mainly characterized in terms of their tensile and flame-retardancy properties [17]. Pandanus amaryllifolius (a member of the Pandanaceae family, abundant in south-east Asian countries) fibers were extracted via a water-retting-extraction process and investigated as a potential fiber reinforcer in a polymer composite [26].

However, the main challenge in the full development of these bio-based compounds, as replacements for traditional-plastic-based formulations, is the lack of good mechanical characteristics, resulting from poor interfacial adhesion between the filler and matrix [13,27,28] or weak miscibility between the two phases in blend preparations [29]. Different strategies should be studied in order to adapt these systems to our needs and improve their efficiency. An alkaline and benzoyl chloride treatment of sugar palm fibers was proposed by Sherwani et al. [13]. The combination of treated sugar palm fiber/glass fiber reinforced PLA hybrid composites resulted in good physical and mechanical properties, especially after the fiber pretreatment. This composite was designed to replace acrylonitrile butadiene styrene (ABS) plastic in motorcycle batteries [13]. A new modifying agent based on polyacrylic-acid-grafted organosolv lignin was synthesized via free-radical copolymerization and used in combination with chitosan fiber in polylactide acid in the study by Tanjung et al. [27]. Urea-treated halloysite nanotubes have been utilized to reinforce epoxidized natural rubber and to improve the overall properties of composites [28]. Additives based on vegetable oil (sunflower, rapeseed, and castor oil) have been synthesized and introduced in PLA/starch blends to improve their miscibility [29].
